# Identification and validation of transcription factor-driven enhancers of genes related to lipid metabolism in metastatic oral squamous cell carcinomas

**DOI:** 10.1186/s12903-022-02157-7

**Published:** 2022-04-15

**Authors:** Liru Zhang, Shuangling Zhao, Yuanhang Liu, Feifei Lv, Xixue Geng

**Affiliations:** 1 Department of Stomatology, Second Hospital of Shijiazhuang, Shijiazhuang, 050000 Hebei China; 2 Department of Stomatology, First Outpatient Department of Hebei Province, Shijiazhuang, 050000 Hebei China; 3 Department of Stomatology, Affiliated Hospital of Hebei University, Baoding, 071000 Hebei China; 4 Department of Pathology, Hebei Provincial Hospital of Traditional Chinese Medicine, 389 Zhongshan East Road, Shijiazhuang, 050011 Hebei China

**Keywords:** Oral squamous cell carcinomas, Metastasis-associated enhancers, Lipid metabolism-related genes, CBFB

## Abstract

**Background:**

The role and mechanisms of lipid metabolism in oral squamous cell carcinomas (OSCC) metastasis have not been clarified. This study aims to identify lipid metabolism-related genes and transcription factors regulated by metastasis-associated enhancers (MAEs) in OSCC.

**Methods:**

Gene Set Enrichment Analysis (GSEA) and Gene Set Variation Analysis (GSVA) were performed for lipid metabolism enrichment. TCGA data were used to analyze the differentially expressed lipid metabolism-related genes. MAEs were analyzed using GSE120634. Overlapping analysis was used to screen the MAE-regulated lipid metabolism-related genes, and the prognosis of these genes was analyzed. Transcription factor prediction was performed for the MAE-regulated lipid metabolism-related genes with prognostic value. Validation of the metastatic specificity of MAEs at ACAT1, OXSM and VAPA locus was performed using GSE88976 and GSE120634. ChIP-qPCR, qRT-PCR and Western blotting were used to verify the regulation of ACAT1, OXSM and VAPA expression by CBFB. Effects of CBFB knockdown on proliferation, invasion and lipid synthesis in metastatic OSCC cells were analyzed.

**Results:**

Lipid metabolism was significantly enhanced in metastatic OSCC compared to non-metastatic OSCC. The expression of 276 lipid metabolism-related genes was significantly upregulated in metastatic OSCC, which were functionally related to lipid uptake, triacylglycerols, phospholipids and sterols metabolism. A total of 6782 MAEs and 176 MAE-regulated lipid metabolism-related genes were filtered. Three MAE-regulated lipid metabolism-related genes, ACAT1, OXSM and VAPA, were associated with a poor prognosis in OSCC patients. Enhancers at ACAT1, OXSM and VAPA locus were metastasis-specific enhancers. CBFB regulated ACAT1, OXSM and VAPA expression by binding to the enhancers of these genes. Knockdown of CBFB inhibited proliferation, invasion and lipid synthesis in metastatic OSCC cells.

**Conclusion:**

The MAE-regulated lipid metabolism-related genes (ACAT1, OXSM and VAPA) and the key transcription factor (CBFB) were identified. CBFB knockdown inhibited proliferation, invasion and lipid synthesis of OSCC cells. These findings provide novel candidates for the development of therapeutic targets for OSCC.

**Supplementary Information:**

The online version contains supplementary material available at 10.1186/s12903-022-02157-7.

## Introduction

Oral cancer is a common malignancy of the head and neck, with oral squamous cell carcinomas (OSCC) being the predominant pathological type, accounting for over 90% of oral cancer cases [1]. Surgery is the first option for OSCC treatment. Treatment of advanced OSCC is usually supplemented with radiotherapy and chemotherapy [2]. Metastasis is an important cause of poor prognosis in OSCC, with 40% of OSCC patients being accompanied by lymph node metastasis [3]. The 5-year survival for OSCC patients without metastases is about 90%, while for patients with metastatic OSCC the 5-year survival is only about 40% [4]. Exploring the molecular mechanisms of OSCC metastasis is crucial for discovering effective clinical intervention strategies.

Abnormal cellular metabolism is closely related to tumor development and metastasis [5, 6]. Lipid metabolism provides energy and material basis for cellular life activities. Lipid metabolites are involved in the establishment of cellular structures (e.g. phospholipids and cholesterol), and in signaling influencing a variety of malignant phenotypes, including proliferation, invasion, migration, stemness and drug resistance [5–8]. Abnormal lipid metabolism promotes tumor development, which in turn affects cellular lipid metabolism [9]. Lipid metabolism of tumor cells is characterized by the enhancement of de novo fatty acid synthesis and lipid synthesis [6, 9]. Reprogramming of lipid metabolism provides tumor cells with energy and cellular building block molecules to satisfy the demand of rapid tumor cell proliferation and metastasis [5]. Increased adipogenesis in OSCC is associated with increasing invasiveness of cells [10, 11]. At present, the regulation of lipid metabolism in OSCC progression is largely unknown [9, 12–14]. Effective regulation of lipid metabolism in OSCC metastasis is expected to be a promising approach for future treatment of OSCC. Hence, it is necessary to explore the molecular mechanisms underlying the regulation of lipid metabolism in OSCC.

The objective of this study was to identify lipid metabolism-related genes regulated by metastasis-associated enhancers (MAEs), and the key transcription factor of these genes in OSCC. This study could provide novel therapeutic targets for OSCC treatment.

## Methods

### Data mining

RNA-seq and clinical data of OSCC patients, including 28 metastatic and 47 non-metastatic OSCC patients, were downloaded from The Cancer Genome Atlas (TCGA, https://tcga-data.nci.nih.gov/tcga/). Metastatic OSCC were defined as the patients with metastatic recurrence within 5 years after surgery. Non-metastatic OSCC were defined as the patients without metastasis within 5 years after surgery. Data was normalized using the “edgeR” package. GSE120634 and GSE88976 datasets were downloaded from the Gene Expression Omnibus (GEO, http://www.ncbi.nlm.nih.gov/geo/).

### Gene set enrichment analysis (GSEA)

GSEA (http://www.broadinstitute.org/gsea/index.jsp) was performed using the data downloaded from TCGA database. The "HALLMARK_ADIPOGENESIS" and "HALLMARK_FATTY_ACID_METABOLISM" gene sets, which were downloaded from the Molecular Signatures Database (MSigDB, http://www.broad.mit.edu/gsea/msigdb/), were selected for lipid metabolism-related studies. Genes in these two gene sets were recognized as lipid metabolism-related genes for subsequent studies. Expression of lipid metabolism-related genes in both metastatic OSCC and non-metastatic OSCC was analyzed. Significant enrichment was screened with normalized (NOM) *P* < 0.05, normalized enrichment score (NES) ≥ 1 and false discovery rate (FDR) q-value ≤ 0.25.

### Gene set variation analysis (GSVA)

RNA-seq data of metastatic OSCC patients from TCGA were used for GSVA. GSVA was performed to calculate adipogenesis, fatty acid metabolism and tumor-associated signaling pathway scores using the "GSVA" package in R. The R package "stats" was used to calculate the Pearson’s correlation among tumor-related signaling pathway scores, adipogenesis score and fatty acid metabolism score. Correlation coefficient > 0 and *P* < 0.05 indicated a significant positive correlation. Tumor-related signaling pathways with significant positive correlation with both adipogenesis score and fatty acid metabolism score were represented in a heatmap.

### Identification and functional analysis of differentially expressed genes related to lipid metabolism

Lipid metabolism-related genes were extracted based on "HALLMARK_ADIPOGENESIS" and "HALLMARK_FATTY_ACID_METABOLISM" gene sets in MSigDB. Differential expression analysis of lipid metabolism-related genes between metastatic OSCC and non-metastatic OSCC was carried out with data from TCGA database using the R package “limma”. Selection criteria for differentially expressed genes were |Log2 fold change|≥ 1.0 and adjusted *P* < 0.05. Functional analysis of lipid metabolism-related differentially expressed genes was performed by Metascape (http://metascape.org/gp/index.html).

### Identification of MAEs and MAE-regulated lipid metabolism-related genes

H3K27ac ChIP-seq data of metastatic OSCC cells (HN120Met) and primary OSCC cells (HN120Pri) from GSE120634 dataset were used for MAEs identification. Significant H3K27ac peaks were identified using “findPeaks” tool in HOMER software. H3K27ac peaks with |fold change|> 4 and *P* < 0.05 in HN120Met versus HN120Pri were considered to be significantly different H3K27ac peaks. Enhancers with H3K27ac signals fold change > 4 in HN120Met compared to HN120Pri were screened as MAEs. The enhancers nearest to lipid metabolism-related genes on the genome were analyzed using the “annotatePeaks” tool in HOMER software. Overlapping analysis was performed using R. GSE120634 and GSE88976 datasets were used for H3K27ac signaling analysis at ACAT1, OXSM and VAPA locus. Visualization of H3K27ac peaks was performed using the Integrative Genomics Viewer (IGV, https://igv.org).

### Prognostic signature identification and transcription factor prediction of MAE-regulated genes related to lipid metabolism

MAE-regulated genes related to lipid metabolism were analyzed for distant metastasis-free survival (DMFS) and overall survival (OS) based on TCGA. OSCC patients were grouped into high- and low-expression groups according to the quartiles. Kaplan–Meier plots and log-rank tests were used for analysis of DMFS and OS. For genes significantly affecting DMFS, the Kaplan–Meier Plotter tool (https://kmplot.com/analysis/) with default parameters was used to analyze relapse-free survival (RFS) and OS. *P* < 0.05 was used for significance identification. Toolkit for Cistrome Data Browser (http://dbtoolkit.cistrome.org) was used for transcription factor prediction. Pearson’s correlation analysis of the expression of transcription factors and genes was performed using the R package "stats".

### Cell culture and transfection

The human metastatic OSCC cells, HSC3 (JCRB Cell Bank, Japan) and BHY (Cell Lines Service, Germany), were cultured in Dulbecco’s modified of Eagle medium (DMEM, Gibco, USA) with 10% fetal bovine serum (FBS, Gibco, USA) and 1% penicillin–streptomycin (Sigma-Aldrich, USA) at 37 °C, 5% CO_2_. Small interfering RNA (siRNA) targeting CBFB (si-CBFB) and negative control (si-NC) were purchased from Gene Pharma (Shanghai, China). HSC3 and BHY cells were seeded in 6-well plates with 1 × 10^6^ cells/well, and transfected with si-CBFB or si-NC using Lipofectamine 3000 (Invitrogen, USA) according to the manufacturer’s instruction.

### Quantitative real-time PCR (qRT-PCR)

Total RNA was extracted using Trizol (Invitrogen, USA). High-Capacity cDNA Reverse Transcription Kit (Applied Biosystems, USA) was used for cDNA synthesis. qRT-PCR amplification reactions were performed using Power SYBRs Green PCR Master Mix (Applied Biosystems, USA) and ABI 7900 real-time PCR system (Applied Biosystems, USA). GAPDH was set as the internal reference gene. Relative expression of genes was calculated using 2^−ΔΔCt^ method. Primers for qRT-PCR were listed as follows: CBFB-F: 5′-AGAAGCAAGTTCGAGAACGAG-3′; CBFB-R: 5′-CCTGAAGCCCGTGTACTTAATCT-3′. ACAT1-F: 5′-AAGGCAGGCAGTATTGGGTG-3′; ACAT1-R: 5′-ACATCAGTTAGCCCGTCTTTTAC-3′. OXSM-F: 5′-TGATGCTGGTCACATAACTGC-3′; OXSM-R: 5′-TCCCAATGGTGTGGAAGTAGC-3′. VAPA-F: 5′-AGATCCTGGTCCTCGATCCG-3′; VAPA-R: 5′-TTTTCTATCCGATGGATTTCGCA-3′. GAPDH-F: 5′-GGAGCGAGATCCCTCCAAAAT-3′; GAPDH-R: 5′-GGCTGTTGTCATACTTCTCATGG-3′.

### Chromatin immunoprecipitation quantitative real time PCR (ChIP-qPCR)

Cells were cross-linked using 1% formaldehyde for 10 min at room temperature, and then lysed using cell lysis solution for 20 min. Lysates were sonicated to fragments of 200–500 bp, and then incubated with H3K27ac antibody (ab4729, Abcam, USA) at 4 °C overnight. ChIP-DNA fragments were purified using the Gel Extraction Kit (Omega Bio-Tek, USA). Enhancers of ACAT1, OXSM and VAPA were used for ChIP-qPCR analysis. The primers were listed as follows: ACAT1-F: 5′- GCCCAGTTCTCAGAGCCATT-3′; ACAT1-R: 5′- GGGAGGGGAGATGGTTTTGG-3′. OXSM-F: 5′- GGAATGAAAGGGGAAGGCGA-3′; OXSM-R: 5′- GTCTAGGCAGTGATGCTCCC-3′. VAPA-F: 5′- GGGCCTCGTTTCAGTCTTCA-3′; VAPA-R: 5′- AGCGCATCACACTCTTCCAA-3′.

### Cell proliferation assay

Cell Counting Kit-8 (CCK-8) (Solarbio, China) was used for cell proliferation analysis according to the manufacturer’s instructions. HSC3 and BHY cells were seeded into 96-well plates with 5 × 10^3^ cells/well, and incubated at 37 °C for 0, 1, 2 and 3 days. 10 µl of CCK-8 reagent was added into each well according to the above time points. A microplate reader (Thermo Scientific, USA) was used to determine the absorbance value at 450 nm.

### Cell invasion assay

For cell invasion analysis, Matrigel (30 µg/well) (Becton Dickinson, USA) was added to Transwell chambers (Greiner Bio-One, Austria). Cells were cultured in serum-free DMEM medium to a density of 1 × 10^5^ cells/ml. 200 µl of cell suspension was added to the upper chamber, and 500 µl of DMEM with 10% FBS was added to the lower chamber. Then, cells were incubated at 37 °C for 48 h. The invaded cells at the bottom of chambers were fixed with 2.5% glutaraldehyde for 10 min, while the non-invaded cells were discarded. The invaded cells were stained with 0.1% crystal violet for 20 min. Five randomly selected visual fields were counted with a light microscope (Carl Zeiss, Germany) at × 200 magnification.

### Western blotting

Total protein was extracted using RIPA buffer (Sigma-Aldrich, USA). Protein concentration was quantified using the Pierce BCA protein assay (Thermo Scientific, USA). Proteins were separated using 10% sodium dodecyl sulfate–polyacrylamide gel electrophoresis (SDS-PAGE), and then transferred to polyvinylidene difluoride membranes (Millipore, USA). After fixed in 5% skimmed milk, proteins were incubated with primary antibodies overnight at 4 °C. Then, the membranes were washed with PBS buffer, and incubated with goat anti-rabbit IgG H&L (1: 5000, ab96899, Abcam, USA) at room temperature for 1 h. β-actin was selected as the internal reference. Protein bands were visualized using Enhanced Chemiluminescence Detection Kit (Amersham Life Science, USA), and analyzed using Image J software. Primary antibodies were as follows: anti-fatty acid synthase (1:2000, ab128870, Abcam, USA), anti-SCD (1:2000, ab236868, Abcam, USA), anti-ACACA (1:2000, ab269273, USA), anti-ACAT1 (1:2000, ab154396, Abcam, USA), anti-OXSM (1:2000, ab229111, Abcam, USA), anti-VAPA (1:2000, ab176995, Abcam, USA), anti-CBFB (1:2000, ab133600, Abcam, USA) and anti-β-actin (1:2000, ab181092, Abcam, USA).

### Triglycerides (TG) and total cholesterol (TC) analysis

HSC3 and BHY cells were seeded in 6-well plates with 5 × 10^5^ cells/well, and then incubated at 37 °C for 18 h. Total cholesterol (TC) assay kit 96 T (Nanjing Jiancheng, China) and Triglycerides (TG) assay kit 96 T (Nanjing Jiancheng, China) were used for the determination of relative cellular TG and TC content according to the manufacturer’s instructions.

### Statistical analysis

Data were presented as mean ± SD. Statistical analysis was performed using R software 3.6 and GraphPad prim 9.1.0. Student's *t*-test was applied for comparison between two groups. One-way ANOVA followed by Tukey’s post hoc test was used for comparison among multiple groups. *P* < 0.05 indicated the significant difference.

## Results

### Lipid metabolism was enhanced in metastatic OSCC, and associated with tumor-related signaling pathways

**Fig. 1:**
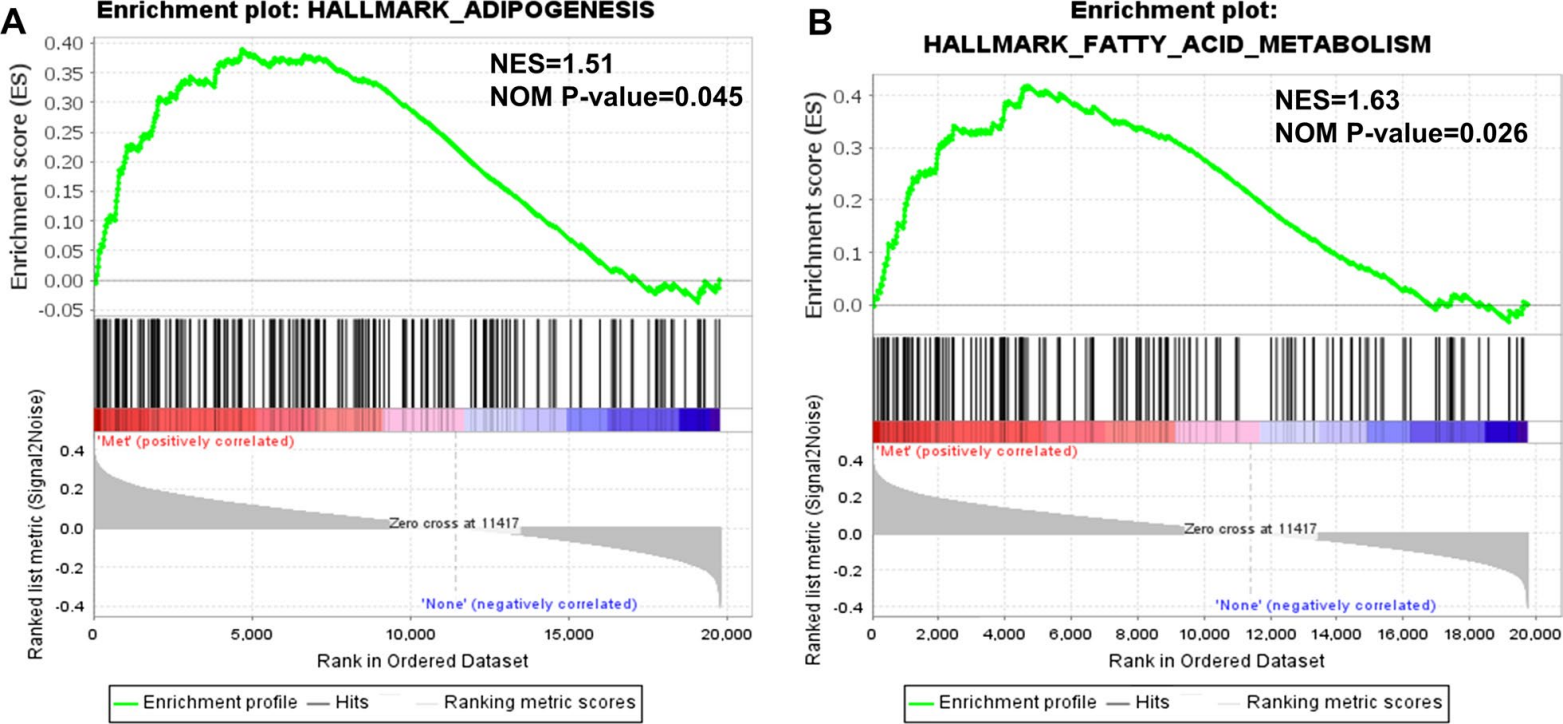
GSEA enrichment plot of "adipogenesis" and "fatty acid metabolism" in metastatic and non-metastatic OSCC. Met, metastatic OSCC. None, non-metastatic OSCC

**Fig. 2:**
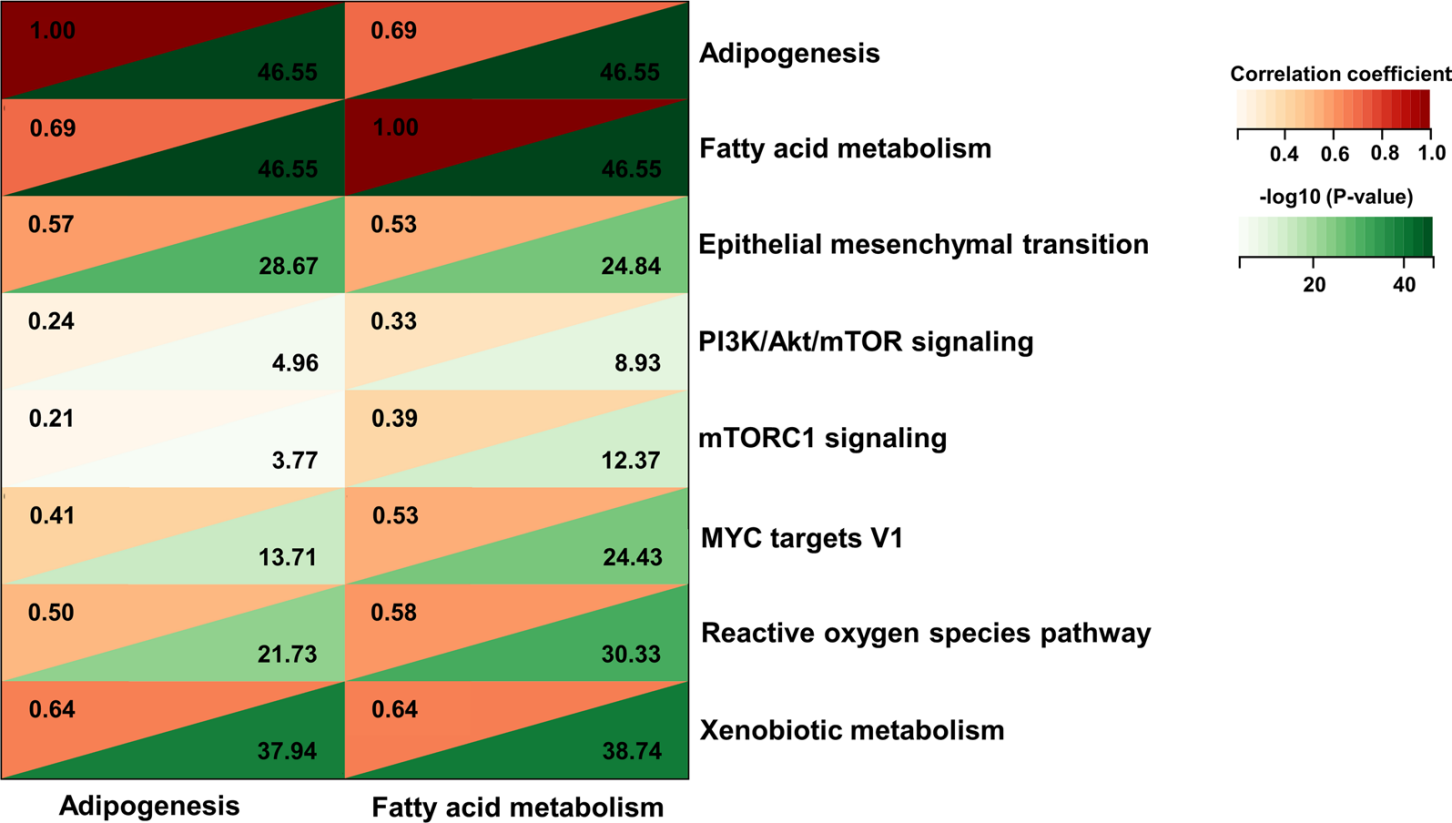
Pearson’s correlation analysis of "fatty acid metabolism", "adipogenesis" and tumor-associated signaling pathway scores in metastatic OSCC. GSVA score was calculated for metastatic OSCC. Tumor-associated signaling pathways that were significantly positively correlated with "adipogenesis" and "fatty acid metabolism" scores were shown in the heatmap

Lipid metabolism reprogramming is an important hallmark of malignancy [15]. To investigate differences in lipid metabolism between metastatic OSCC and non-metastatic OSCC, we extracted two gene signatures, "fatty acid metabolism" and "adipogenesis", in MSigDB database. GSEA showed that the expression levels of genes from "adipogenesis" and "fatty acid metabolism" signatures were significantly higher in metastatic OSCC than non-metastatic OSCC (Fig. 1A, B). Subsequently, GSVA was performed on metastatic OSCC samples. A total of six tumor-related signaling pathways (containing “epithelial mesenchymal transition”, “PI3K/Akt/mTOR signaling”, “mTORC1 signaling”, “MYC targets V1”, “reactive oxygen species pathway” and “xenobiotic metabolism”) were significantly and positively correlated with both "fatty acid metabolism" and "adipogenesis" (Fig. 2). Taken together, these results showed that lipid metabolism was enhanced in metastatic OSCC compared with non-metastatic OSCC, and lipid metabolism showed a positive correlation with tumor-related signaling pathways in metastatic OSCC.

### Identification and functional analysis of differentially expressed genes related to lipid metabolism

**Fig. 3:**
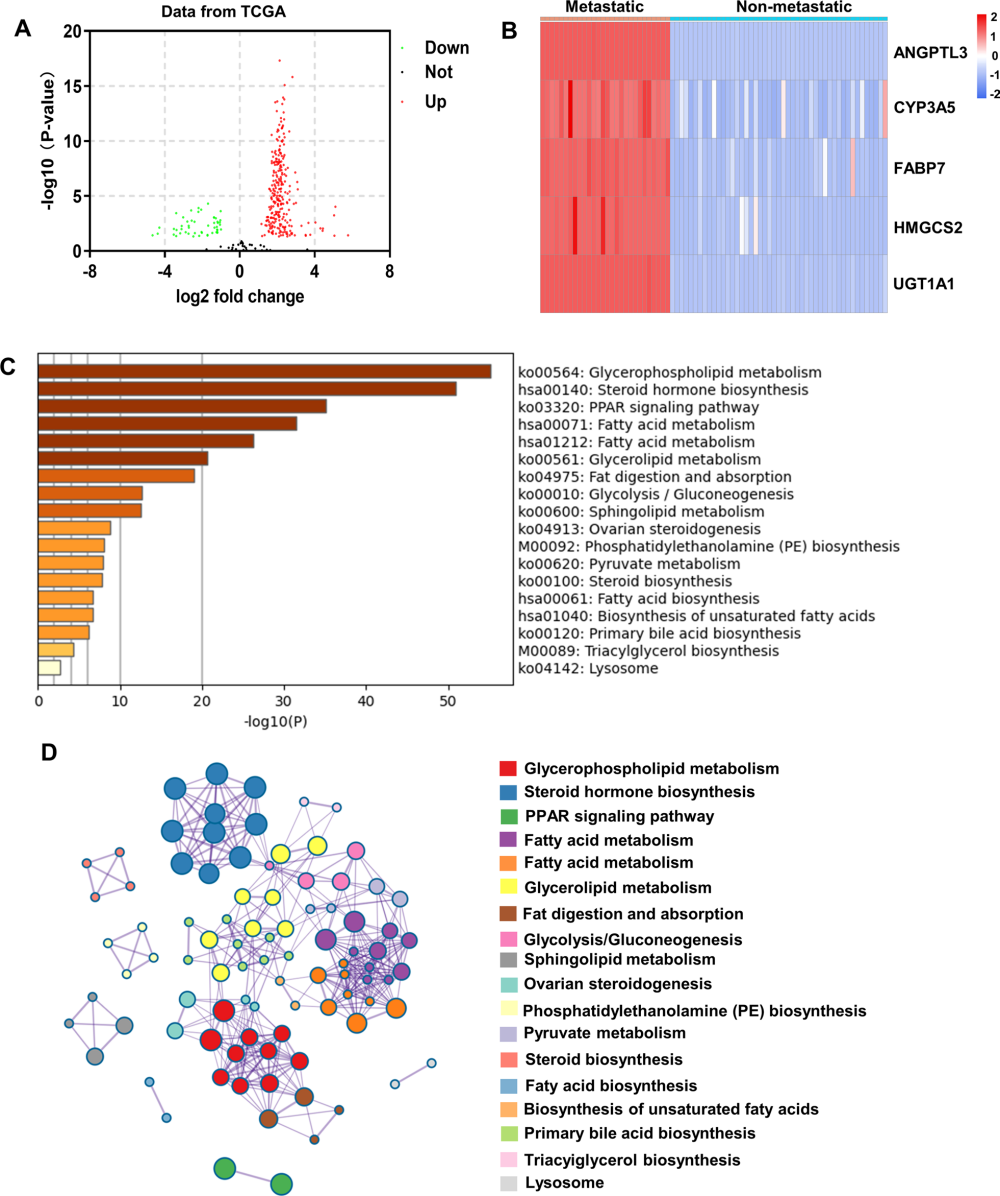
Identification and functional analysis of differentially expressed genes related to lipid metabolism. **A** Volcano plot of differentially expressed genes in metastatic OSCC compared to non-metastatic OSCC. Thresholds were set as *P* < 0.05 and |log2 fold change|> 1. Red circle indicated upregulated genes in metastatic OSCC. Green circle indicated downregulated genes in metastatic OSCC. Grey circle indicated genes with no significant difference between metastatic and non-metastatic OSCC. **B** Heatmap of the top five upregulated genes in metastatic OSCC versus non-metastatic OSCC. **C** Boxplot of functional enrichment of upregulated genes. **D** Network of upregulated genes enrichment terms. Circles with the same color represented the same biological process. Size of circle was positively correlated with the number of genes contained in the term. Terms with a similarity score above 0.3 were connected by edges, with wider edges corresponding to higher similarity score

To further investigate the differences in lipid metabolism between metastatic and non-metastatic OSCC, the expression of 353 genes from the "adipogenesis" and "fatty acid metabolism" signatures was analyzed. Compared with non-metastatic OSCC, there were 276 upregulated genes, 51 downregulated genes and 26 non-differentially expressed genes in metastatic OSCC (Fig. 3A). The 276 upregulated genes were ranked according to log2 fold change in descending order. The top five upregulated genes, ANGPTL3, CYP3A5, FABP7, HMGCS2 and UGT1A1, were shown in Fig. 3B. Subsequently, the 276 upregulated genes were functionally analyzed by Metascape. The upregulated genes were enriched in “glycerophospholipid metabolism”, “fatty acid metabolism”, “glycerolipid metabolism”, “fat digestion and absorption”, “sphingolipid metabolism”, “Phosphatidylethanolamine (PE) biosynthesis”, “fatty acid biosynthesis”, “biosynthesis of unsaturated fatty acids”, “steroid hormone biosynthesis”, “ovarian steroidogenesis”, “steroid biosynthesis”, “primary bile acid biosynthesis” and “triacylglycerol biosynthesis” (Fig. 3C). The upregulated genes were also involved in PPAR signaling pathway and the metabolism of glycolysis, gluconeogenesis and pyruvate (Fig. 3C). Additionally, there was a strong similarity among the enriched biological processes (Fig. 3D). Collectively, a total of 276 lipid metabolism-related genes were upregulated in metastatic OSCC compared to non-metastatic OSCC, and these genes were associated with a variety of biological processes related to lipid metabolism.

### Analysis of the prognostic MAE-regulated genes related to lipid metabolism

**Fig. 4:**
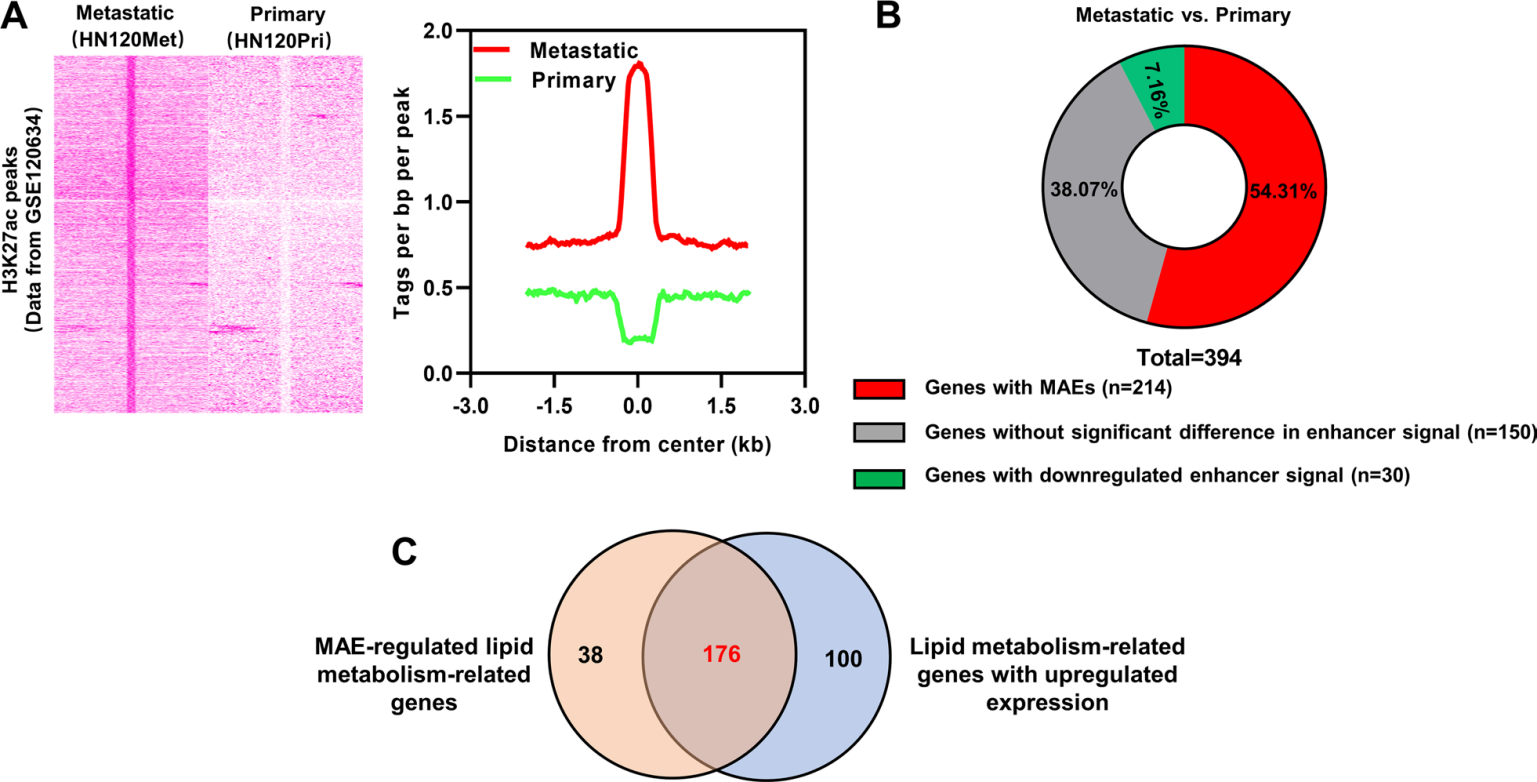
Identification of MAE-regulated lipid metabolism-related genes. **A** Heatmap and aggregation plots of H3K27ac signaling in metastatic OSCC and primary OSCC cells. **B** Distribution of lipid metabolism-related genes corresponding to changes in enhancer signaling in metastatic OSCC versus primary OSCC cells. **C** Overlapping of the potential MAE-regulated lipid metabolism-related genes (n = 214) and the lipid metabolism-related genes with upregulated expression in metastatic OSCC (n = 276)

To screen the MAE-regulated genes, we analyzed H3K27ac ChIP-seq data of HN120Met (metastatic OSCC cell line) and HN120Pri (primary OSCC cell line) which were obtained from GSE120634 dataset. Enhancers with > fourfold upregulation of H3K27ac signaling in HN120Met cells versus HN120Pri cells were defined as MAEs. A total of 6782 MAEs were identified (Fig. 4A). Furthermore, we found 214 lipid metabolism-related genes with MAEs, 30 lipid metabolism-related genes with downregulated enhancer signals (> fourfold downregulation of H3K27ac signaling) and 150 lipid metabolism-related genes without significant change in enhancer signals in HN120Met cells compared to HN120Pri cells (Fig. 4B). A total of 176 MAE-regulated genes associated with lipid metabolism were screened by taking intersections of upregulated lipid metabolism-related genes (n = 276) and lipid metabolism-related genes with MAEs (n = 214) (Fig. 4C).

**Fig. 5:**
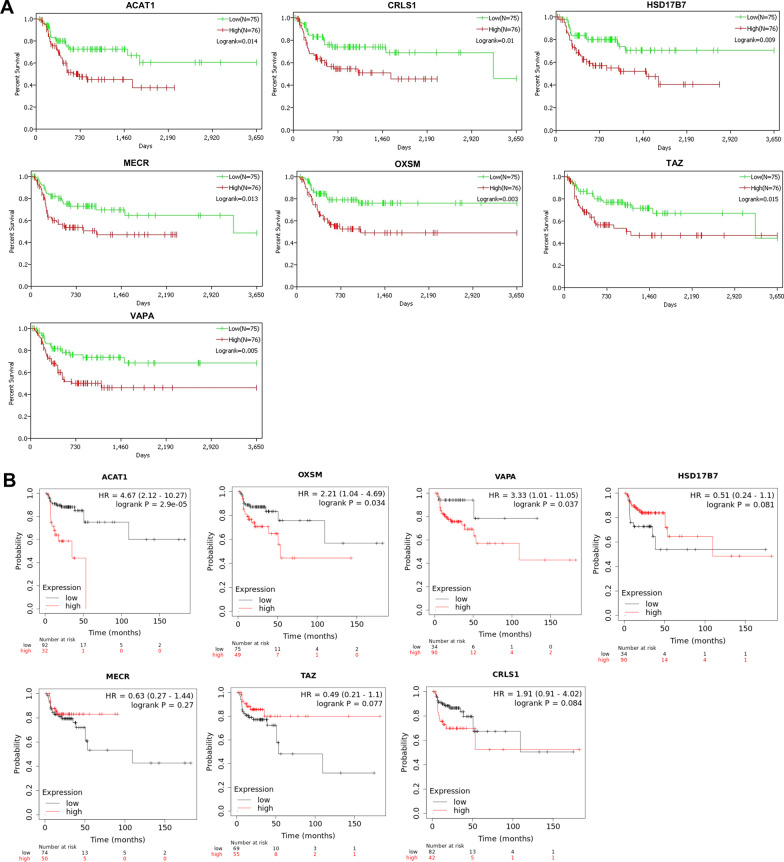
Screening of MAE-regulated lipid metabolism-related genes with high expression corresponding to a poor prognosis. **A** TCGA based analysis screened the high expression of ACAT1, CRLS1, HSD17B7, MECR, OXSM, TAZ and VAPA corresponding to a poor DMFS. Patients were divided into high- and low-expression groups according to the quartiles. **B** Kaplan–Meier Plotter tool with default parameters was used to analyze the effects of ACAT1, CRLS1, HSD17B7, MECR, OXSM, TAZ and VAPA expression on RFS of OSCC patients. Patients were grouped using the “auto select best cutoff”

Next, we assessed the impacts of 176 MAE-regulated lipid metabolism-related genes on DMFS of OSCC patients using TCGA data. High expression of 7 genes (ACAT1, CRLS1, HSD17B7, MECR, OXSM, TAZ and VAPA) was corresponded to a dismal DMFS (Fig. 5A). Kaplan–Meier Plotter tool was used to analyze RFS of the above 7 genes. Results showed that high expression of ACAT1, OXSM and VAPA corresponded to a poor RFS, while the expression of CRLS1, HSD17B7, MECR and TAZ had no significant effect on RFS in OSCC patients (Fig. 5B). Furthermore, high expression of ACAT1, OXSM and VAPA had adverse effects on OS of OSCC patients, although the effects of ACAT1 and OXSM were not significant (Additional file 1: Fig. S1; Additional file 2: Fig. S2). Taken together, we screened 176 MAE-regulated lipid metabolism-related genes, of which high expression of ACAT1, OXSM and VAPA was associated with a poor prognosis of OSCC patients.

### Prediction of transcription factors for ACAT1, OXSM and VAPA

**Fig. 6:**
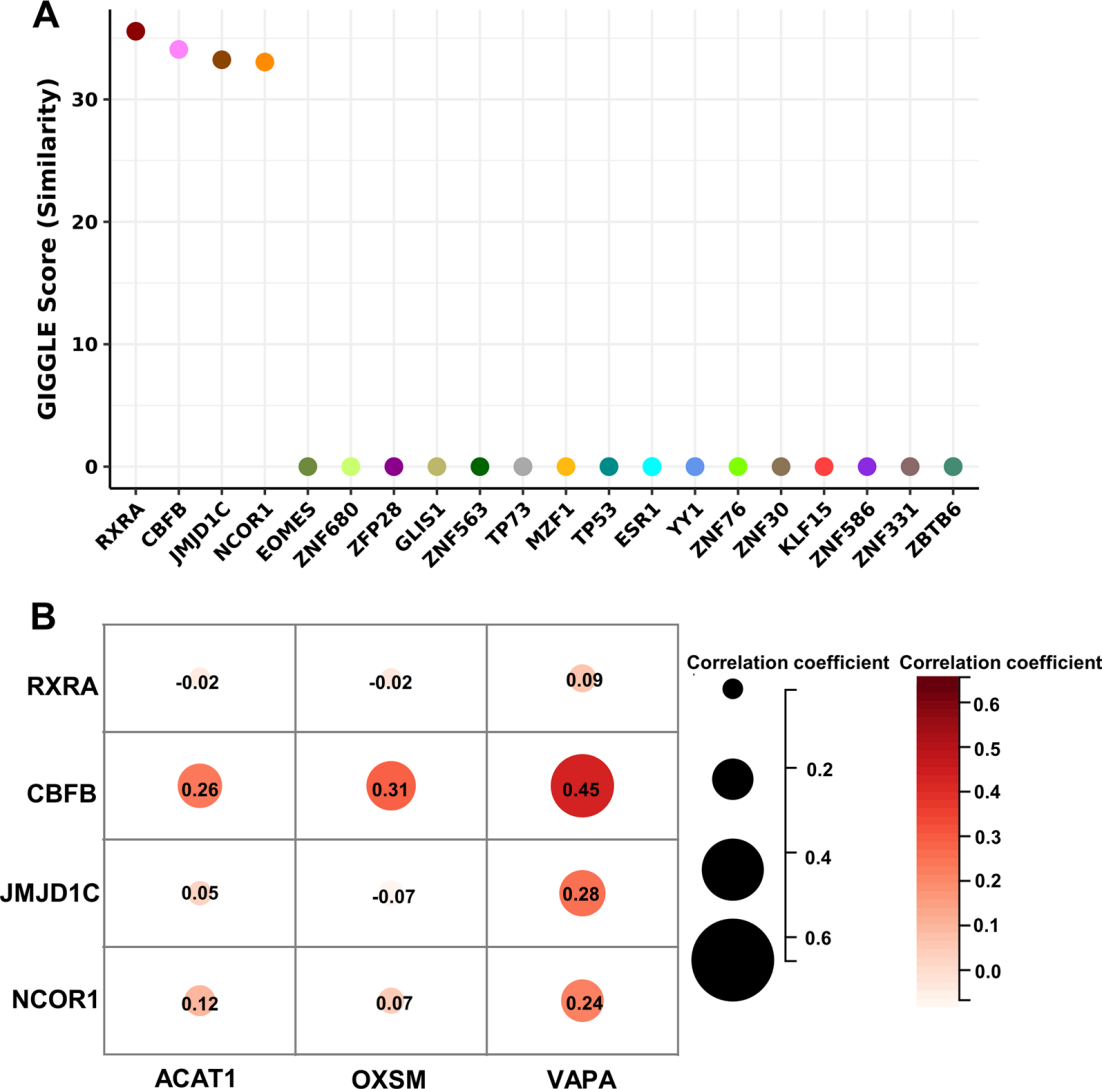
Prediction and expression correlation analysis of transcription factors for ACAT1, OXSM and VAPA. A, GIGGLE score of 20 predicted transcription factors. B, Pearson’s correlation analysis of RXRA, CBFB, JMJD1C, NCOR1, ACAT1, OXSM and VAPA expression based on TCGA database

Toolkit for Cistrome Data Browser (http://dbtoolkit.cistrome.org) was used to predict the transcription factors for ACAT1, OXSM and VAPA. Twenty potential transcription factors were obtained (Fig. 6A). However, the GIGGLE scores of EOMES, ZNF680, ZFP28, GLIS1, ZNF563, TP73, MZF1, TP53, ESR1, YY1, ZNF76, ZNF30, KLF15, ZNF586, ZNF331 and ZBTB6 were zero (Fig. 6A). Therefore, we selected RXRA, CBFB, JMJD1C and NCOR1 for expression correlation analysis. The expression levels of CBFB and NCOR1 were positively correlated with the levels of ACAT1, OXSM and VAPA (Fig. 6B). Especially, CBFB showed the highest correlation coefficient with ACAT1, OXSM and VAPA (Fig. 6B). Therefore, CBFB was selected for the following study.

### CBFB driven ACAT1, OXSM and VAPA expression by binding enhancers

**Fig. 7:**
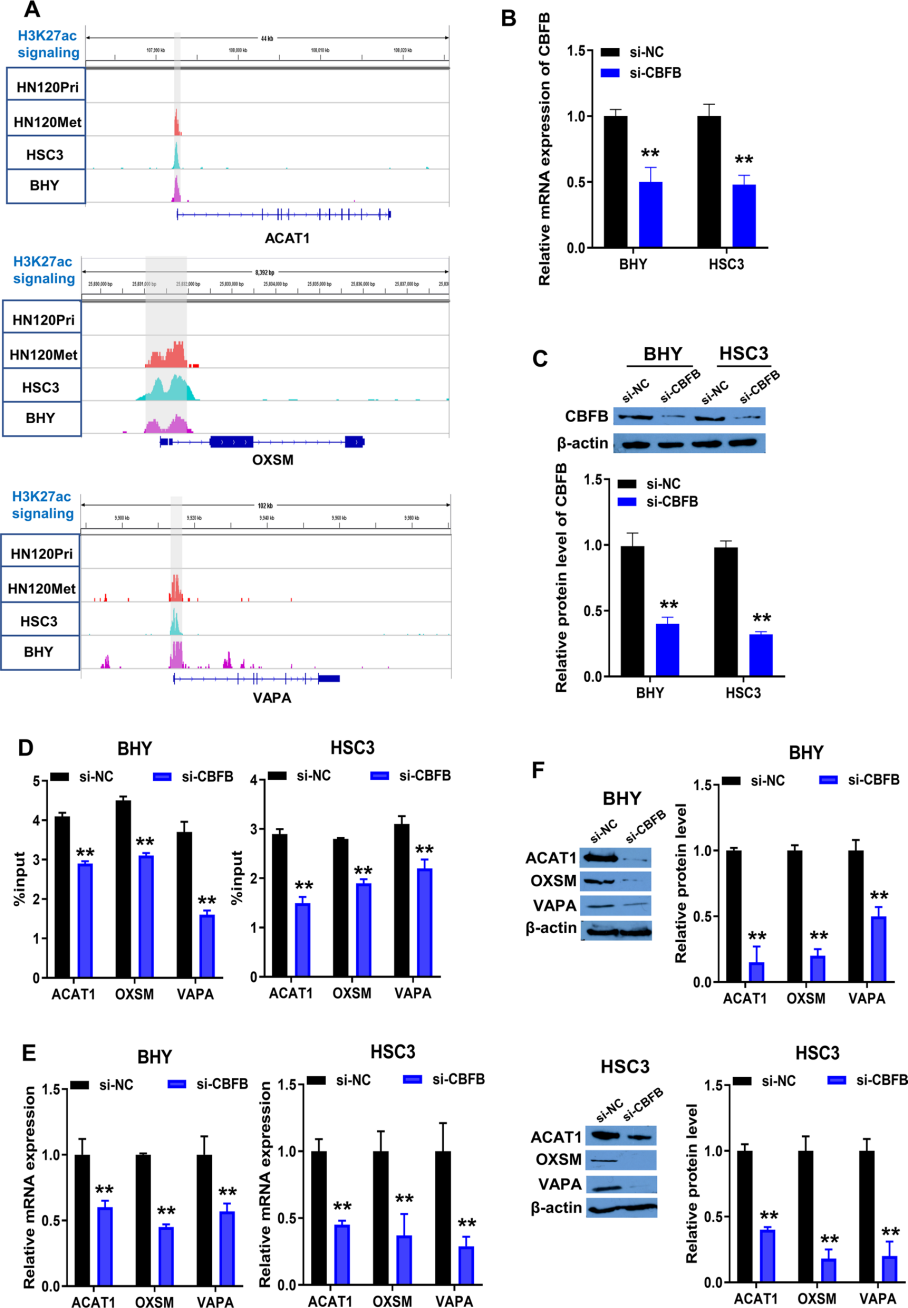
Regulation of ACAT1, OXSM and VAPA expression by CBFB in metastatic OSCC cells. **A** Genome regions with enhanced H3K27ac signaling at ACAT1, OXSM and VAPA locus in metastatic OSCC cells (HN120Met, BHY and HSC3 cells) and primary OSCC cell (HN120Pri cell) were analyzed using GSE88976. **B**/**C** qRT-PCR and Western blotting were used to measure the mRNA (**B**) and protein (**C**) levels of CBFB in BHY and HSC3 cells transfected with si-NC or si-CBFB. **D** ChIP-qPCR was performed to detect the alterations in H3K27ac modification of enhancers of ACAT1, OXSM and VAPA after CBFB knockdown in BHY and HSC3 cells. **E**/**F** qRT-PCR and Western blotting were performed to detect the effects of CBFB knockdown on mRNA (**E**) and protein (**F**) expression of ACAT1, OXSM and VAPA in BHY and HSC3 cells. All of the experiments were performed with three-time independently. **P < 0.001, si-CBFB versus si-NC

To explore whether CBFB drives the expression of ACAT1, OXSM and VAPA through binding enhancers, we analyzed the distribution of genes and enhancers in the genome. Conserved H3K27ac signaling enriched regions were present around the locus of ACAT1, OXSM and VAPA in metastatic OSCC cells (HN120Met, BHY and HSC3), which were absent in primary OSCC cells (HN120Pri) (Fig. 7A).

BHY and HSC3 cells were transfected with si-CBFB or si-NC. The knockdown status of CBFB was detected by qRT-PCR and Western blotting post 3 days of si-CBFB or si-NC treatment. Transfection with si-CBFB significantly inhibited CBFB mRNA and protein expression in BHY and HSC3 cells (Fig. 7B, C; Additional file 3: Fig. S3A), indicating that CBFB knockdown cells were successfully constructed.

Effect of CBFB knockdown on H3K27ac modification of enhancers of ACAT1, OXSM and VAPA was examined using ChIP-qPCR. H3K27ac levels of enhancers of ACAT1, OXSM and VAPA were significantly reduced in si-CBFB group compared to si-NC group (Fig. 7D). Additionally, mRNA and protein expression levels of ACAT1, OXSM and VAPA were significantly reduced upon CBFB knockdown in BHY and HSC3 cells (Fig. 7E, F; Additional file 3: Fig. S3B, C). Taken together, knockdown of CBFB inhibited the activation of ACAT1, OXSM and VAPA enhancers, and inhibited the expression of ACAT1, OXSM and VAPA in vitro.

### Knockdown of CBFB inhibited proliferation, invasion and lipid synthesis of metastatic OSCC cells

**Fig. 8:**
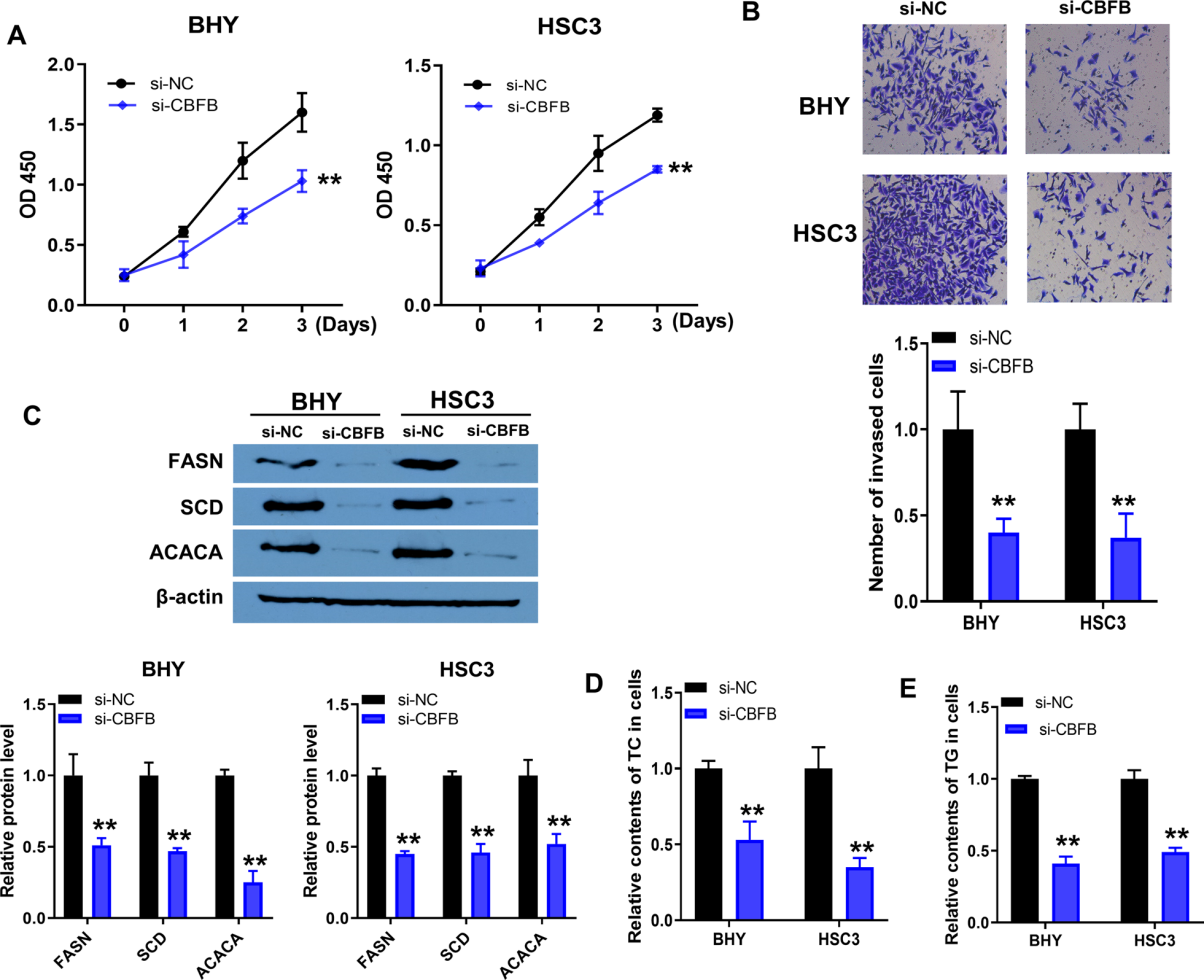
Knockdown of CBFB inhibited proliferation, invasion and lipid synthesis of metastatic OSCC cells. A/B, CCK8 (**A**) and Transwell (**B**) assays were used to analysis the effects of CBFB knockdown on proliferation and invasion of BHY and HSC3 cells. **C** Protein expression of FASN, SCD and ACACA in BHY and HSC3 cells with si-CBFB or si-NC transfection. **D**/**E** TC (**D**) and TG (**E**) levels in BHY and HSC3 cells upon CBFB knockdown. All of the experiments were performed with three-time independently. **P < 0.001, si-CBFB versus si-NC. TC, total cholesterol. TG, triglyceride

To investigate the effects of CBFB on the malignant phenotype of OSCC cells, the proliferation of BHY and HSC3 cells upon CBFB knockdown was assessed using CCK8. Knockdown of CBFB impaired the proliferation of BHY and HSC3 cells (Fig. 8A). Transwell results showed that knockdown of CBFB significantly inhibited the invasion of BHY and HSC3 cells (Fig. 8B). Aberrant activation of de novo lipid synthesis is one of the key characteristics that distinguishes tumor cells from normal cells, as de novo lipid synthesis is rare in normal cells, whereas the majority (~ 90%) of fatty acids in tumor cells are formed by de novo synthesis [16, 17]. ACACA, FASN and SCD are key enzymes in de novo lipid synthesis [15, 18]. Therefore, in the present study we focused on the effects of CBFB knockdown on the expression of ACACA, FASN and SCD. Compared to si-NC group, knockdown of CBFB significantly downregulated the expression of FASN, SCD and ACACA in BHY and HSC3 cells (Fig. 8C; Additional file 3: Fig. S3D). The cellular TC and TG levels of si-CBFB group were lower than si-NC group (Fig. 8D, E). Taken together, these results suggested that knockdown of CBFB inhibited the proliferation, invasion and lipid synthesis of OSCC cells.

## Discussion

Prognosis of OSCC is poor due to the occurrence of metastasis [1, 19, 20]. Development and metastasis of OSCC are influenced by a variety of factors, among which metabolic factors have attracted increasing attention [21–24]. In this study, we identified the MAE-regulated lipid metabolism-related genes and the key transcription factor in OSCC. Furthermore, the function of the key transcription factor was verified in vitro.

Lipids are important components of cell structure, and play pivotal roles in many biological processes such as cell signaling, immune response and energy provision [5–8]. In contrast to normal cells, tumor cells are usually characterized by significantly higher lipid uptake and synthesis [5, 6, 9]. Particularly, normal cells typically do not undergo de novo lipid synthesis, whereas approximately 90% of fatty acids are de novo synthesized in tumor cells [16, 17]. Studies have shown that abnormal lipid metabolism is directly related to the development and metastasis of malignant tumors [25, 26]. Wu et al. found that OSCC progression is associated with lipid metabolism reprogramming, and three lipid metabolism-related genes, TGFB1, SPP1 and SERPINE1, are independent prognostic factors for OSCC [9]. It has been shown that bitter melon extract treatment inhibits adipogenesis and induces apoptosis in OSCC cells [27]. However, studies on lipid metabolism in metastasis of OSCC are still rare. In this study, we analyzed adipogenesis and fatty acid metabolism in metastatic OSCC versus non-metastatic OSCC by GSEA, and found that genes related to lipid metabolism were significantly enriched in metastatic OSCC, suggesting that lipid metabolism is involved in metastasis in OSCC. Lipid metabolism was significantly and positively correlated with epithelial mesenchymal transition (EMT), PI3K/Akt/mTOR signaling, mTOR complex 1 (mTORC1) signaling, MYC targets V1, reactive oxygen species (ROS) pathway and xenobiotic metabolism. EMT is an important initiating process in the metastasis of tumor cells, allowing epithelial cells to lose apical-basal polarity and cell–cell adhesion, and acquire mobility and invasiveness [28, 29]. PI3K/AKT/mTOR pathway regulates a variety of cellular processes including growth, proliferation, metastasis and radiosensitivity [30–32]. MYC has pro-carcinogenic effects in OSCC, such as promoting proliferation, invasion and migration of OSCC cells [33, 34]. These evidences further suggest that lipid metabolism was enhanced in metastatic OSCC, and in turn promotes OSCC metastasis.

To further investigate lipid metabolism in OSCC, we analyzed the expression of 353 lipid metabolism-related genes in metastatic and non-metastatic OSCC. A total of 276 genes were screened for upregulation in metastatic OSCC, representing 78.12% of the total number of all lipid metabolism-related genes. This result suggested that the expression of the majority of lipid metabolism-related genes was enhanced in metastatic OSCC. Lipids include three major categories, including triacylglycerols, sterols and phospholipids [35]. The function of upregulated genes in metastatic OSCC has been analyzed in more detail. We found that genes upregulated in metastatic OSCC were significantly enriched in multiple pathways related to lipid uptake, fatty acid synthesis, phospholipid metabolism, sterol metabolism and triacylglycerol biosynthesis. These results suggested that OSCC metastasis was associated with multiple lipid metabolic pathways that encompass the three major classes of lipids.

Following the identification of upregulated genes associated with lipid metabolism in metastatic OSCC, we wished to investigate the underlying mechanisms of upregulated expression of these genes. Enhancers is a class of DNA *cis*-acting elements that promote the transcriptional activity of target genes [36]. In tumor cells, aberrant activation of enhancer regulates the expression of oncogenes and tumor suppressor genes [36–38]. Herein, we expect to screen the MAEs and lipid metabolism-related genes regulated by MAEs. Upregulation of H3K27ac levels is a significant hallmark of enhancer activation. We screened 214 potential MAE-regulated lipid metabolism-related genes, of which the expression of 176 was significantly upregulated in metastatic OSCC. DMFS of 176 MAE-regulated lipid metabolism-associated genes was analyzed, of which the expression of 7 genes corresponded to a poor DMFS. Further analysis of RFS of these 7 genes resulted in 3 genes, ACAT1, OXSM and VAPA, that significantly affected the prognosis of OSCC. In addition, patients with high expression of ACAT1, OXSM and VAPA had a poor OS. Acetyl-CoA acetyltransferase 1 (ACAT1) catalyzes the reversible formation of acetoacetyl-CoA from two molecules of acetyl-CoA. Studies have demonstrated that ACAT1 promotes fatty acid synthase (FASN) stabilization, thereby promoting lipid metabolism and hepatocarcinogenesis [39]. Small molecule ACAT1 inhibitors have potential anticancer effects [40]. 3-oxoacyl-ACP synthase, mitochondrial (OXSM), also known as FASN2D, is associated with the elongation of fatty acid chains in mitochondria [41]. VAMP associated protein A (VAPA) encodes a type IV membrane protein that plays a role in cell motility and vesicular transport, etc [42]. Kyong-Ah Yoon et al. found that the fusion gene VAPA-Rab31 promotes proliferation and metastasis of lung cancer cells by upregulating Bcl-2 and activating CREB [43]. However, studies on the role and mechanism of ACAT1, OXSM and VAPA in OSCC have not been well characterized.

*Cis*-regulation of enhancers on target genes dependents on the role of transcription factors [44]. In this study, we screened out a key transcription factor, core-binding factor subunit beta (CBFB), that was most strongly associated with the expression of ACAT1, OXSM and VAPA. Abnormal expression of CBFB is associated with blood cancers, breast cancer, colorectal cancer and gastric cancer [45–48]. Marie Bleakley et al. demonstrated that the expression of CBFB-MYH11 fusion protein facilitates T cell recognition and acute myeloid leukemia cells death [45]. CBFB acts as a tumor suppressor in breast cancer [47]. In colorectal cancer, CBFB deletion enhances cell resistance to MEK inhibitors [46]. However, we have not found any studies of CBFB in OSCC development and metastasis. In the present study, we demonstrated that H3K27ac signaling at ACAT1, OXSM and VAPA locus were specifically enhanced in metastatic OSCC cells. Furthermore, we verified that CBFB bound to enhancers of ACAT1, OXSM and VAPA, and demonstrated that CBFB knockdown resulted in a downregulation of ACAT1, OXSM and VAPA expression. These results confirmed that CBFB was a transcription factor for MAE-regulated lipid metabolism-related genes. Knockdown of CBFB inhibited proliferation and invasion of metastatic OSCC cells. FASN, searoyl-CoA desaturase (SCD) and acetyl-CoA carboxylase alpha (ACACA) are key genes in de novo lipid synthesis [49–51]. In particular, ACACA is the rate-limiting enzyme for fatty acid synthesis [49, 51]. Knockdown of CBFB inhibited the expression of FASN, SCD and ACACA, and reduced cellular TC and TG levels, suggesting that knockdown of CBFB inhibited lipid synthesis in metastatic OSCC cells. Effects and underlying mechanisms of CBFB and its target genes on metabolism and metastasis of OSCC still needed to be intensively investigated in the future.

## Conclusion

In this study, we identified three MAE-regulated lipid metabolism-related genes (ACAT1, OXSM and VAPA). CBFB was a key transcription factor for ACAT1, OXSM and VAPA in metastatic OSCC cells. Knockdown of CBFB inhibited proliferation, invasion and lipid synthesis in metastatic OSCC cells. ACAT1, OXSM, VAPA and CBFB have the potential to become the therapeutic targets for OSCC.

## Supplementary Information

**Additional file 1.** **Fig. S1.** Effects of ACAT1, CRLS1, HSD17B7, MECR, OXSM, TAZ and VAPA expression on the overall survival of OSCC patients based on TCGA data.

**Additional file 2.** **Fig. S2.** Effects of ACAT1, CRLS1, HSD17B7, MECR, OXSM, TAZ and VAPA expression on the overall survival was analyzed using Kaplan-Meier Plotter tool.

**Additional file 3.** **Fig. S3.** Original images of Western blotting. A, original images of Western blotting for Fig. 7C. Protein levels of CBFB in BHY and HSC3 cells transfected with si-NC or si-CBFB. β-actin was selected as the internal control. B/C, original images of Western blotting for Fig. 7F. Protein levels of ACAT1, OXSM and VAPA in BHY and HSC3 cells transfected with si-NC or si-CBFB. β-actin was selected as the internal control. D, original images of Western blotting for Fig. 8C. Protein levels of FASN, SCD and ACACA in BHY and HSC3 cells with si-NC or si-CBFB transfection. β-actin was selected as the internal control.

## Data Availability

The datasets generated and/or analysed during the current study are available in the TCGA (https://tcga-data.nci.nih.gov/tcga/) and GEO (GSE120634 and GSE88976) repository.
