# Supplementary figures and images for: Identification and validation of transcription factor-driven enhancers of genes related to lipid metabolism in metastatic oral squamous cell carcinomas
Source: BMC Oral Health. 2022 Apr 15;22:126. doi: 10.1186/s12903-022-02157-7 (PMC9013160; doi:10.1186/s12903-022-02157-7)

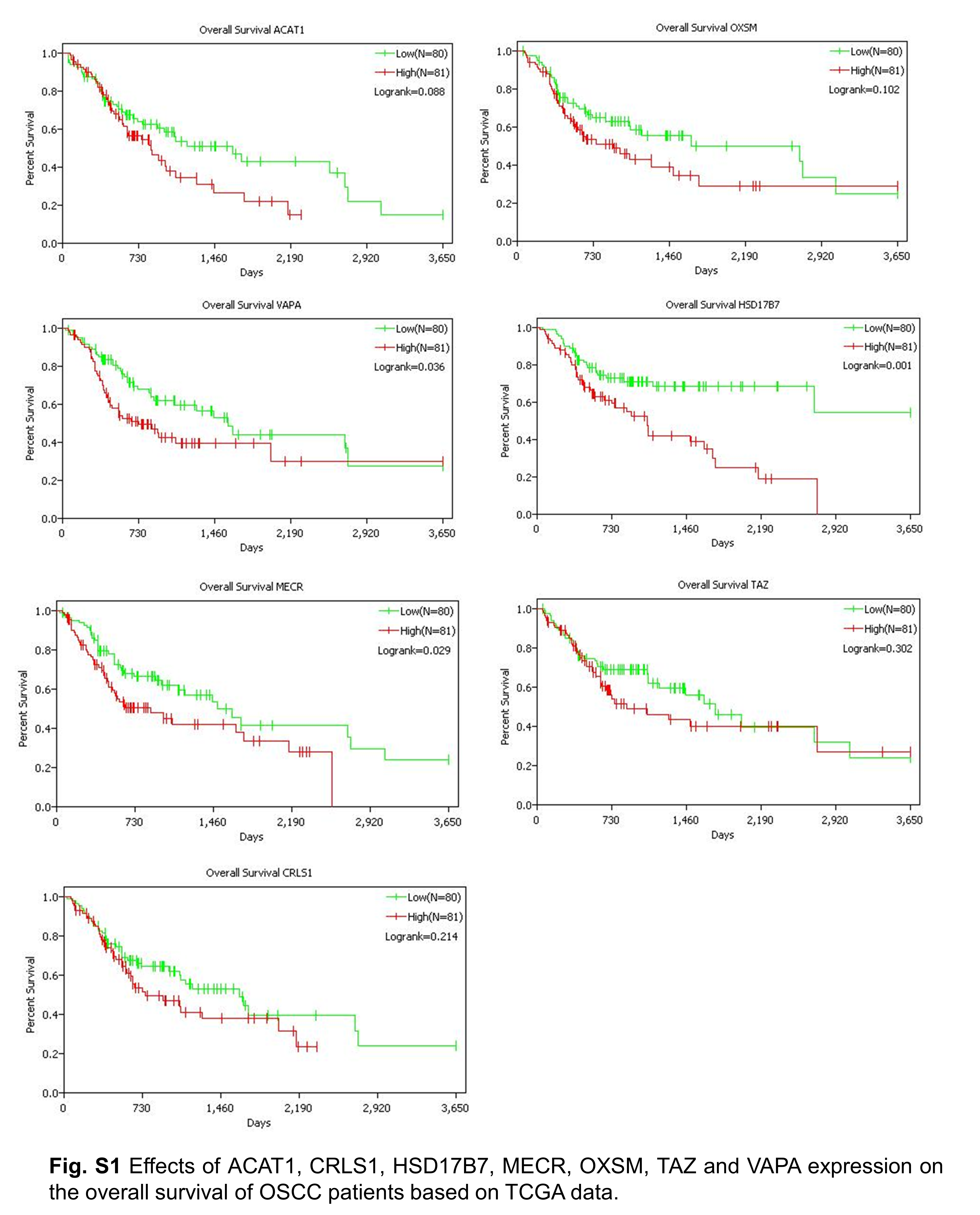

Supplement: Supplementary file 1 — Additional file 1. Fig. S1. Effects of ACAT1, CRLS1, HSD17B7, MECR, OXSM, TAZ and VAPA expression on the overall survival of OSCC patients based on TCGA data. [file 12903_2022_2157_MOESM1_ESM.tif]

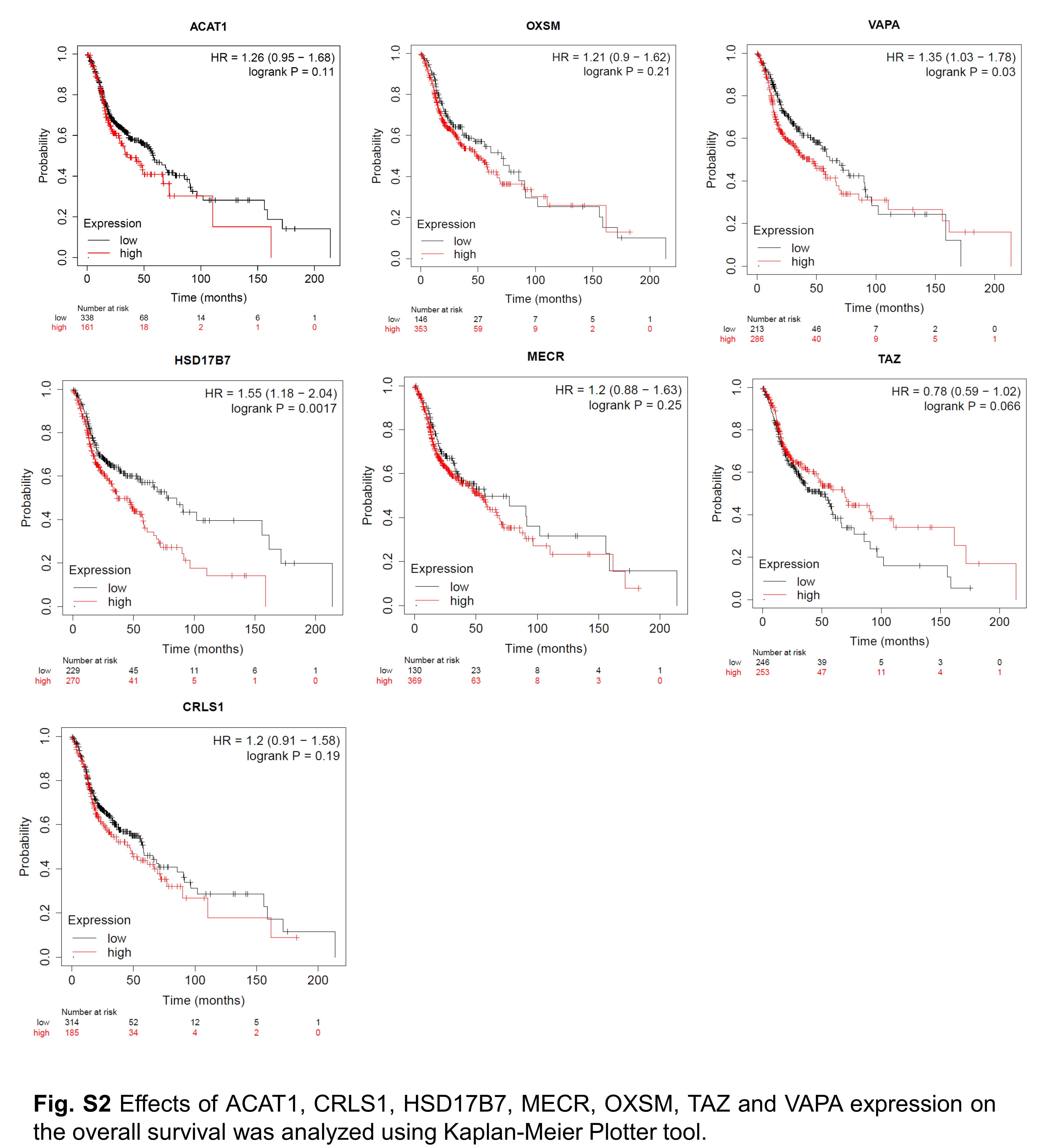

Supplement: Supplementary file 2 — Additional file 2. Fig. S2. Effects of ACAT1, CRLS1, HSD17B7, MECR, OXSM, TAZ and VAPA expression on the overall survival was analyzed using Kaplan-Meier Plotter tool. [file 12903_2022_2157_MOESM2_ESM.tif]

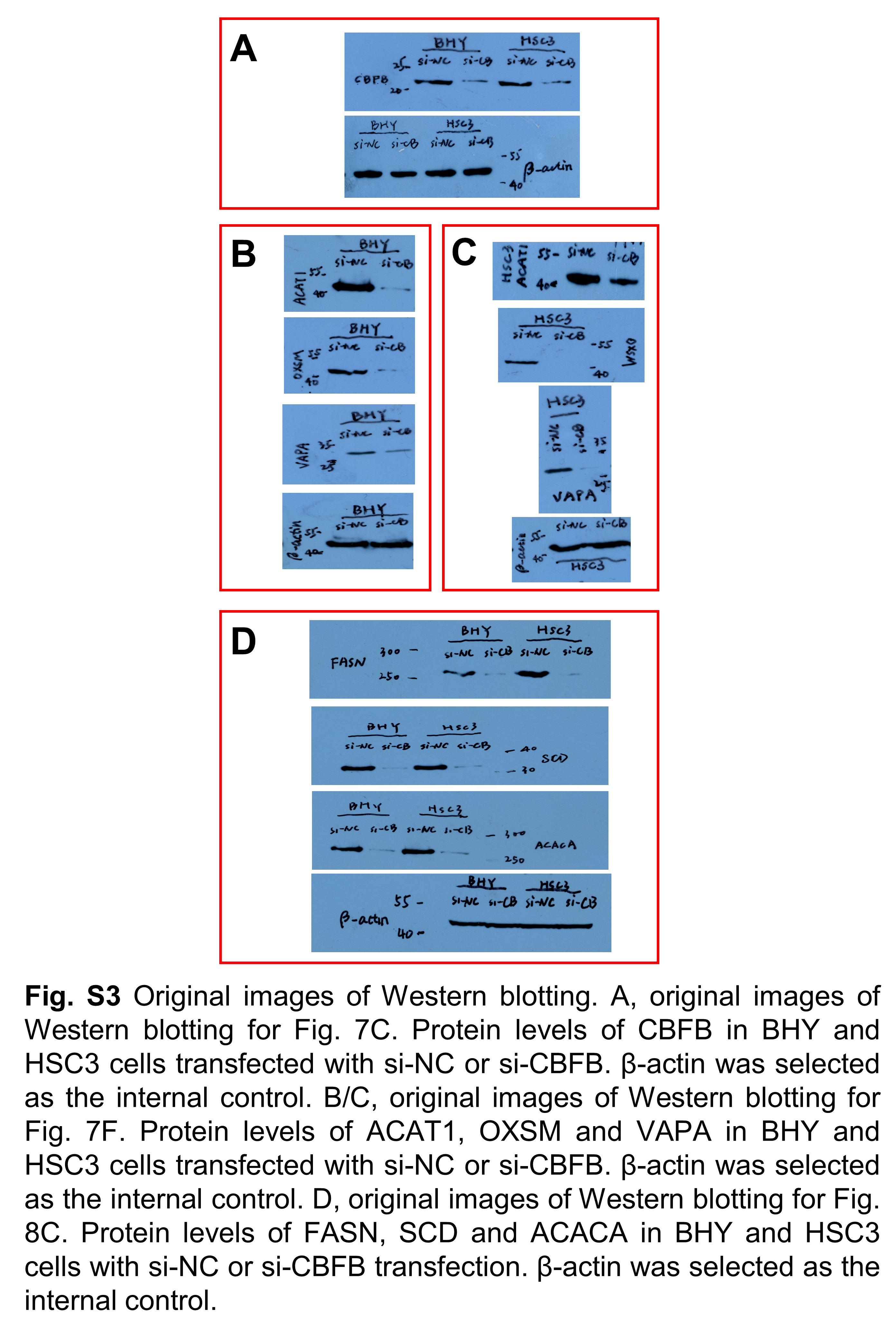

Supplement: Supplementary file 3 — Additional file 3. Fig. S3. Original images of Western blotting. A, original images of Western blotting for Fig. 7C. Protein levels of CBFB in BHY and HSC3 cells transfected with si-NC or si-CBFB. β-actin was selected as the internal control. B/C, original images of Western blotting for Fig. 7F. Protein levels of ACAT1, OXSM and VAPA in BHY and HSC3 cells transfected with si-NC or si-CBFB. β-actin was selected as the internal control. D, original images of Western blotting for Fig. 8C. Protein levels of FASN, SCD and ACACA in BHY and HSC3 cells with si-NC or si-CBFB transfection. β-actin was selected as the internal control. [file 12903_2022_2157_MOESM3_ESM.tif]
